# Guidance for biostatisticians on their essential contributions to clinical and translational research protocol review

**DOI:** 10.1017/cts.2021.814

**Published:** 2021-07-12

**Authors:** Jody D. Ciolino, Cathie Spino, Walter T. Ambrosius, Shokoufeh Khalatbari, Shari Messinger Cayetano, Jodi A. Lapidus, Paul J Nietert, Robert A. Oster, Susan M. Perkins, Brad H. Pollock, Gina-Maria Pomann, Lori Lyn Price, Todd W. Rice, Tor D. Tosteson, Christopher J. Lindsell, Heidi Spratt

**Affiliations:** 1 Department of Preventive Medicine, Division of Biostatistics, Feinberg School of Medicine, Northwestern University, Chicago, IL, USA; 2 Department of Biostatistics, University of Michigan, Washington Heights, Ann Arbor, MI, USA; 3 Department of Biostatistics and Data Science, Division of Public Health Sciences, Wake Forest School of Medicine, Winston-Salem, NC, USA; 4 Michigan Institute for Clinical & Health Research (MICHR), University of Michigan, Ann Arbor, MI, USA; 5 Department of Public Health, Division of Biostatistics, University of Miami, Miami, FL, USA; 6 School of Public Health, Oregon Health & Sciences University, Portland, OR, USA; 7 Department of Public Health Sciences, Medical University of South Carolina, Charleston, SC, USA; 8 Department of Medicine, Division of Preventive Medicine, University of Alabama at Birmingham, Birmingham, AL, UK; 9 Department of Biostatistics, Indiana University, Indianapolis, IN, USA; 10 Department of Public Health Sciences, UC Davis School of Medicine, Davis, CA, USA; 11 Duke Biostatistics, Epidemiology and Research Design (BERD) Methods Core, Duke University, Durham, NC, USA; 12 Tufts Clinical and Translational Science Institute, Tufts University, Boston, MA, USA; 13 Institute of Clinical Research and Health Policy Studies, Tufts Medical Center, Boston, MA, USA; 14 Department of Medicine, Division of Allergy, Pulmonary, and Critical Care Medicine, Medical Director, Vanderbilt Human Research Protections Program, Vice-President for Clinical Trials Innovation and Operations, Nashville, TN, USA; 15 Department of Biomedical Data Science, Division of Biostatistics, Geisel School of Medicine at Dartmouth, Hanover, NH, USA; 16 Department of Biostatistics, Vanderbilt University Medical Center, Nashville, TN, USA; 17 Department of Preventive Medicine and Population Health, University of Texas Medical Branch, Galveston, TX, USA

**Keywords:** Protocol, Review, Biostatistician, Scientific rigor, Translational research

## Abstract

Rigorous scientific review of research protocols is critical to making funding decisions, and to the protection of both human and non-human research participants. Given the increasing complexity of research designs and data analysis methods, quantitative experts, such as biostatisticians, play an essential role in evaluating the rigor and reproducibility of proposed methods. However, there is a common misconception that a statistician’s input is relevant only to sample size/power and statistical analysis sections of a protocol. The comprehensive nature of a biostatistical review coupled with limited guidance on key components of protocol review motived this work. Members of the Biostatistics, Epidemiology, and Research Design Special Interest Group of the Association for Clinical and Translational Science used a consensus approach to identify the elements of research protocols that a biostatistician should consider in a review, and provide specific guidance on how each element should be reviewed. We present the resulting review framework as an educational tool and guideline for biostatisticians navigating review boards and panels. We briefly describe the approach to developing the framework, and we provide a comprehensive checklist and guidance on review of each protocol element. We posit that the biostatistical reviewer, through their breadth of engagement across multiple disciplines and experience with a range of research designs, can and should contribute significantly beyond review of the statistical analysis plan and sample size justification. Through careful scientific review, we hope to prevent excess resource expenditure and risk to humans and animals on poorly planned studies.

## Introduction

Rigorous scientific review of research protocols is critical to making funding decisions [1, 2], and to the protection of both human and non-human research participants [3]. Two pillars of ethical clinical and translational research include scientific validity and independent review of the proposed research [4]. As such, the review process often emphasizes the scientific approach and the study design, along with rigor and reproducibility of data collection and analysis. The criterion score labeled “Approach” has been shown to be the strongest predictor of the overall Impact Score and the likelihood of funding for research project grants (e.g., R01s) at the National Institutes of Health (NIH) [5]. Evidence also favors scientific review as a consequential component of institutional review of human participant research [3]. Given the increasing complexity of research designs and data analysis methods, quantitative experts, such as biostatisticians, often play an essential role in evaluating the rigor and reproducibility of proposed analytic methods. However, the structure and components of formal review can vary greatly when quantitative methodologists review research protocols prior to data collection, whether for Institutional Review Boards (IRBs), scientific review committees, or intramural and extramural grant review committees.

Protocol submitters and protocol reviewers often mistakenly view a statistician’s input as relevant only to sample size/power and statistical analysis sections of a protocol. Experienced reviewers know that to provide informative and actionable review of a research protocol from a biostatistical perspective requires a comprehensive view of the research strategy. This can be a daunting task to novice quantitative methodologists, yet to our knowledge, there is little guidance on the role and crucial components of biostatistical review of a protocol before data are collected.

Members of the Biostatistics, Epidemiology, and Research Design (BERD) Special Interest Group (SIG) of the Association for Clinical and Translational Science (ACTS) sought to develop this guidance. We used a consensus approach to identify the elements of research protocols that a biostatistician should consider in a review, and provide specific guidance on how each element should be reviewed. The resulting review framework can be used as an educational tool and guideline for biostatisticians navigating review boards and panels. This article briefly describes the approach to developing the framework, provides a comprehensive checklist, and guidance on review of each protocol element. We are disseminating this framework to better position biostatisticians to (1) advocate for research protocols that achieve the goal of answering their proposed study questions while minimizing risk to participants, and (2) serve as a steward of resources, with the ultimate goal of preventing the pursuit of uninformative or unnecessary research activities. We hope a consequence of this work will also be improved rigor and reproducibility of research protocols at the time of submission because protocol writers will also benefit from the guidance.

### Approach to Developing Guidelines

In fall of 2017, the BERD SIG of the ACTS identified the considerable variation in the expectations for, and practice of, biostatistical review of research protocols as a modifiable barrier to effectively informing funding decisions, and to weighing risks and benefits for research participants. The BERD SIG is comprised of biostatisticians and epidemiologists with expertise in clinical and translational research at academic medical centers across the USA. Volunteers from this group formed a working group, consisting of all 16 coauthors of this article, to develop a checklist of items a quantitative methodologist should review in a research protocol (Table 1). The initial checklist focused on defining essential elements for reviewing a randomized controlled trial (RCT) as this is considered the most robust design in clinical research [6]. However, RCTs are not necessarily always feasible, practical, scientifically, or ethically justified, so elements for reviewing other important types of studies were added. Protocol elements essential for an RCT may be irrelevant to other types of studies, and vice versa.

**Table 1.** Checklist guide of items to consider in biostatistical review of protocols

1. Objectives and hypotheses
□ (a) Objectives articulated and consistent: **S**pecific, **M**easurable, **A**chievable, **R**elevant, **T**ime bound
□ (b) Hypotheses follow from objectives
□ (c) Statistical hypothesis tests are clear or easily inferred and match aims
2. General approach
□ (a) General study design matches the objectives and hypotheses to address research question
□ (b) Limitations on conclusions that can be drawn are evident and clear
3. Population and sample
□ (a) Degree of generalizability is obvious
□ (b) Inclusion and exclusion criteria are appropriate for state of knowledge
□ (c) Screening and enrollment processes minimize bias and do not restrict diversity
4. Measurements and outcomes
□ (a) Choice of measurements, especially the response variable, is justified and consistent with the objectives
□ (b) Timing of assessments and measurements is clear and standardized (study schedule or visit matrix should be present)
□ (c) Objectively measured and standardized
□ (d) If based on subjective or patient report, use validated instruments as appropriate
□ (e) Measurements are of maximum feasible resolution with no unnecessary categorization in data collection
□ (f) Ranges of outcomes, distributional properties, and handling in analyses are clear
□ (g) Algorithms used to derive variables or score outcome assessments are justified (e.g., citations, clinical meaning, etc.)
□ (h) Measurement of important/standard explanatory variables that will describe sample or address confounding
5. Treatment assignment
□ (a) Minimization of biases (e.g., randomization and blinding)
□ (b) Control condition(s) allow for comparability or minimization of confounding
6. Data integrity and data management
□ (a) Data capture and management platform is described
□ (b) Security and control of access to study data are discussed
□ (c) Data validation, error corrections, and query resolution processes are included
7. Statistical analysis plan
□ (a) Statistical approach is consistent with hypothesis and objectives
□ (b) A plan for describing the dataset is given
□ (c) Unit of analysis is clearly described for each analysis
□ (d) Analysis populations clearly described (e.g., intention-to-treat set, per protocol set, full analysis set)
□ (e) Key statistical assumptions are addressed
□ (f) Alternative approaches in the event of violations of assumptions are present
□ (g) Discussion of control of type I error (multiple comparisons) is present
□ (h) Description of preventing and handling missing data is given
□ (i) Interim analyses and statistical stopping guidelines are clear and justified
8. Sample size justification
□ (a) Type I and II error rates present for all sample size calculations and corresponding statistical tests
□ (b) Parameter assumptions are clearly stated and justified (i.e., based on previous research and consider the population studied)
□ (c) Statistical tests used in sample size calculations match those presented in statistical analysis plan or appropriately justify reasoning for straying from it
□ (d) Minimum clinically important differences or required precision described
9. Reporting and reproducibility
□ (a) Plans for data sharing and archiving are present
□ (b) Version control or a means of ensuring rigor, transparency, reproducibility in any processes is evident
□ (c) Plan to report results according to guidelines or law

As the checklist was finalized, working group members were assigned to draft guidance describing review essentials pertaining to each item on the checklist. The expectation was that the text should describe the biostatical review perspective arrived at during the group discussions that occurred during development of the checklist. Another assigned reviewer then revised each section. The consensus approach involved multiple iterations of review and revision, and the final text presented in this article reflects the consensus of the working group. The co-primary (Ciolino and Spino) and senior authors (Lindsell and Spratt) synthesized all feedback from revision and review to finalize this article and corresponding checklist. Consensus was reached when all coauthors agreed with the final resultant article and checklist tool.

**Fig. 1.**
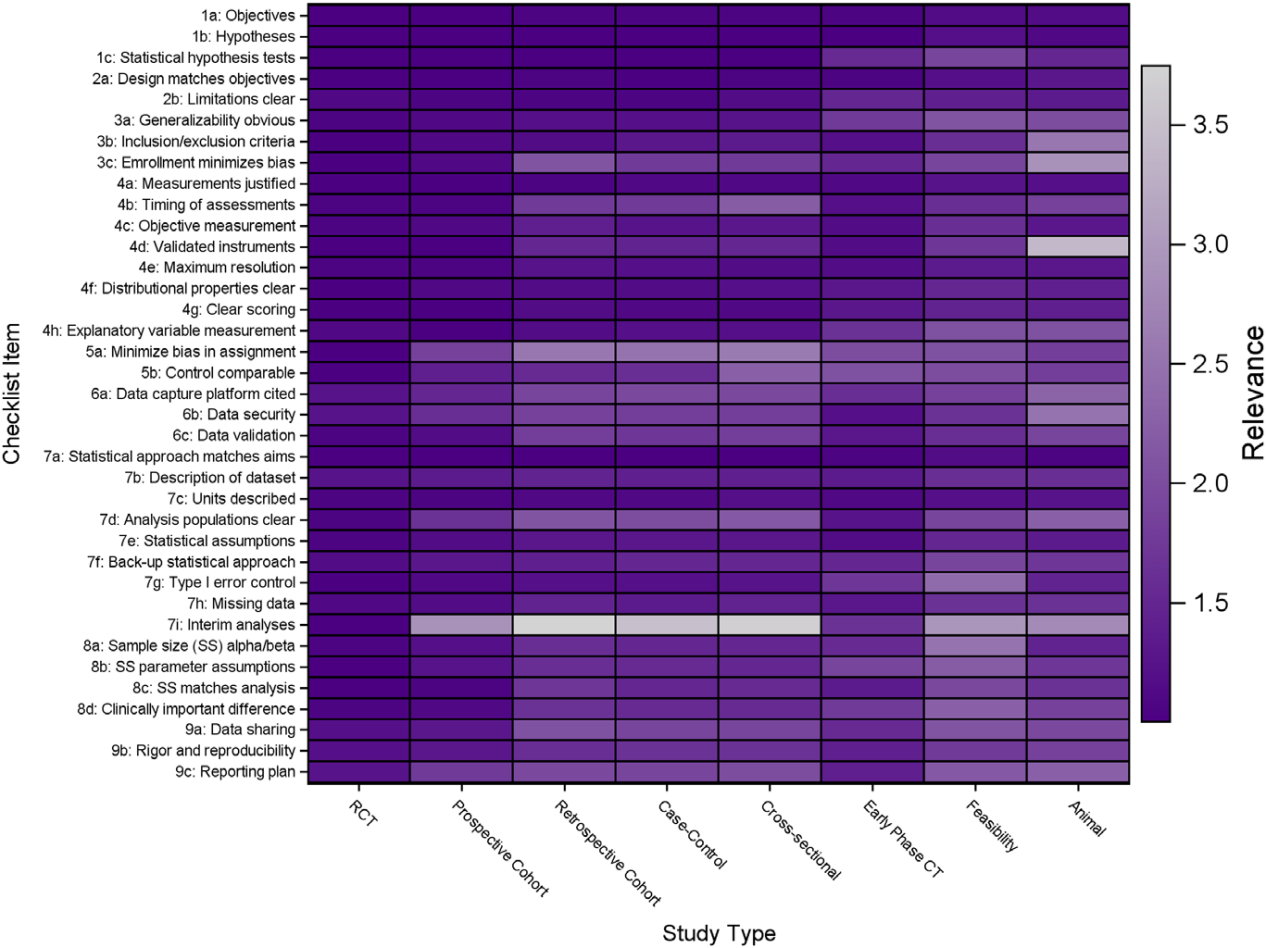
Illustration of varying degrees of relevance for protocol items across common study types. This figure supplements the accompanying checklist of protocol items a biostatistical reviewer should consider in reviewing study protocols. The heat map illustrates the high-level summary view, among coauthors and other quantitative methodologists (*N* = 20 respondents), of relevance for each checklist item. Individual respondents rated each item from 1 (most relevance) to 4 (no relevance/not applicable). Darker cells correspond to higher importance or relevance for a given item/study type, while lighter cells indicate less relevance or importance. If we use the randomized controlled trial (RCT) as a benchmark, we note that the majority of the checklist items are important to consider and review in a research protocol for this study type. The ordering of study types from left to right reflects the order in which respondents were presented these items when completing the survey. The dark column to the left illustrates this. As the study type strays from the RCT, we illustrate the varying degrees of relevance for each of these items. For example, a statistical reviewer should not put weight on things like interim analyses for several of these other study types (cohort studies, case-control, etc.), and the group determined that the use of validated instruments and minimizing bias in enrollment in animal studies are less relevant. On the other hand, the need for clear objectives and hypotheses is consistent throughout, no matter what the study type.

In the spring of 2019, a group of early career investigators (i.e., recipients of K awards) reviewed and provided comment on the checklist and article during a question-and-answer review lunch. Their feedback was that to maximize dissemination and impact beyond the statistical community, it would be more effective to emphasize why the statistical perspective matters for a protocol element rather than trying to justify one statistical argument or another. To obtain additional feedback to help focus the manuscript, we invited members and affiliates of the BERD SIG to rate the relative importance of each protocol element for different study designs (Fig. 1). This figure supplements the accompanying checklist of protocol items a biostatistical reviewer should consider in reviewing study protocols. The heat map illustrates the high-level summary view, among coauthors and other quantitative methodologists, of relevance for each checklist item. Individual respondents (*N* = 20) rated each item from 1 (most relevance) to 4 (no relevance/not applicable). Darker cells correspond to higher importance or relevance for a given item/study type, while lighter cells indicate less relevance or importance. If we use the RCT as a benchmark, we note that the majority of the checklist items are important to consider and review in a research protocol for this study type. The dark column to the left illustrates this. As the study type strays from the RCT, we illustrate the varying degrees of relevance for each of these items. For example, a statistical reviewer should not put weight on things like interim analyses for several of these other study types (cohort studies, case-control, etc.), and the group determined that use of validated instruments and minimizing bias in enrollment in animal studies are less relevant. On the other hand, the need for clear objectives and hypotheses is consistent throughout, no matter what the study type. With this rich context and feedback, we finalized the guidance and checklist for presentation here.

## Objectives and Hypotheses

### Objectives Are Articulated and Consistent: Specific, Measurable, Achievable, Relevant, Time Bound (i.e., SMART)

The first step in protocol review is understanding the research question. Objectives describe the explicit goal(s) of the study and should be clearly stated regardless of design. It is common for the objectives to be summarized in the form of “specific aims.” They should be presented in the context of the broader program of research, including a description of existing knowledge gaps and future directions. Objectives should be written so that they are easily understandable by all who read the protocol.

It is challenging to evaluate the rigor and impact of a study when objectives are diffuse. A common guide to writing objectives is the “SMART” approach [7]. That is, objectives should be **specific** as to exactly what will be accomplished. They should be **measurable** so that it can be determined whether the goals are accomplished. They should be **achievable** within the time, resource, and design constraints. They should be **relevant** to the scientific context and existing state of knowledge. Finally, objectives need to be tied to a specific **time** frame, often the duration of a project funding period. Biostatistical reviewers should evaluate objectives according to these criteria, as it will make them better positioned to properly evaluate the rest of the protocol.

### Hypotheses Follow from Objectives

Hypotheses are statements of expected findings from the research outlined in the objectives. A study can have one or many hypotheses, or none at all. If a study is not designed to test the veracity of some assumed truth, it is not necessary – and often detrimental – to force a hypothesis statement. The biostatistical reviewer should appropriately temper criticism of studies that are “hypothesis generating” as opposed to formal statistical hypothesis testing. The observational, hypothesis generating loop of the scientific method provides an opportunity for the biostatistical reviewer to focus on evaluating the rigor and reproducibility of the proposed work in the absence of a formally testable hypothesis.

### Statistical Hypothesis Tests Are Clear and Match Aims

When a hypothesis is appropriate, it should be stated in a testable framework using the data generated by the proposed study. The biostatistical reviewer should assess how the statistical approach relates to the hypothesis and contextualized by the objectives. Our experience is that an objective with more than one or two key hypotheses has insufficient focus to allow for a rigorous, unbiased study design accompanied by a robust analytic approach. Inclusion of several supportive hypotheses is of less concern.

These same notions of cohesion between objective and analyses apply for preliminary and pilot studies. The objectives of preliminary studies should be clearly stated. They may seek to demonstrate a specific procedure can be performed, a specified number of subjects can be enrolled in a given time frame, or that a technology can be produced. A pilot study with an objective to estimate effect size should be redesigned with alternative objectives because the sample size often precludes estimating the effect size with meaningful precision [8]. The moniker of pilot study is often mistakenly used to justify an underpowered study (i.e., uninformative study) [9]. While it is important that pilot studies specify any hypothesis to be tested in a subsequent definitive study, in general they should seldom (if ever) propose to conduct statistical hypothesis tests [8, 10]. In every case, the biostatistical reviewer should look for objectives that are specific to demonstration or estimation.

## General Approach

### General Study Design Matches the Objectives and Hypotheses to Address Research Questions

Once a study’s purpose is clear, the next goal of a biostatistical review is to confirm the general approach (i.e., type of study) matches the objectives and is consistent with the hypotheses that will be tested. RCTs are generally accepted for confirming causal effects, but there are many situations where they are not feasible nor ethically justified, and well-designed observational (non-experimental) studies are required. For example, RCTs to evaluate parachute use in preventing death and major trauma in a gravitational challenge do not exist because of clear ethical concerns. Between the experimental and observational approaches lie a class of studies called quasi-experimental studies that evaluated interventions or exposures without randomization using design and analytical techniques such as instrumental variables (natural experiments) and propensity scores [11, 12]. The biostatistical reviewer should consider the relevant merits and tradeoffs between the experimental, non-experimental, and quasi-experimental approaches and comment on the strength of evidence for answering the study question.

We highlighted a few possible design approaches in Fig. 1. Within each, there are innumerable design options. For example, with the RCT design, there are crossover, factorial, dose-escalating, and cluster-randomized designs, and many more [6]. The biostatistical reviewer should acknowledge the balance between rigor and feasibility, noting that the most rigorous design may not be the most efficient, least invasive, ethical, or resource preserving.

### Limitations on Conclusions that Can Be Drawn Are Evident and Clear

When using innovative designs, the biostatistical reviewer must consider whether the design was selected because it is most appropriate rather than other factors such as current trends and usage in the field. There are typically multiple designs available to answer similar questions, but the protocol must note the limitations of the design proposed and justify its choice over alternative strategies. As Freidman, Furberg, DeMets, et al. note, “There is no such thing as a perfect study” [6].

When a protocol requires novel or atypical designs, it is imperative that the biostatistician’s review carefully considers potential biases and the downstream analytic implications the designs may present. For example, a dose-finding study using response-adaptive randomization will not allow for conclusions to be drawn regarding drug efficacy in comparison to placebo using classical statistical methods. It will, however, allow for estimation of a maximum tolerated dose for use in later phase studies. This imposes additional responsibilities on the biostatisticians to understand the state of the science within the field of application, the conclusions one can draw from the proposed research and their impact on subsequent studies that build upon the knowledge gained.

## Population and Sample

### Degree of Generalizability Is Obvious

We must recognize that every sample will have limits to generalizability; that is, there will be inherent biases in study design and sampling. RCTs have limits to generalizability as they require specification of eligibility criteria to define the study sample. The more restrictive these criteria are, the less generalizable the inferences become. This concept of generalizability becomes particularly important as reviewers evaluate fully translational research that moves from “bench to bedside.” Basic science and animal studies (i.e., “bench research”) occur in comparatively controlled environments, usually on samples with minimal variability or heterogeneity. The generalizability of these pre-clinical findings to heterogeneous, clinical populations in these situations is limited. For this reason, an effect size observed in pre-clinical populations cannot be generalized to that which one would expect in a clinical population.

All sample selection procedures have advantages and disadvantages, which must be considered when assessing the feasibility, validity, and interpretation of study findings. Biases may be subtle, yet they can have important implications for the interpretability and generalizability of study findings. For example, a randomized, multicenter study conducted in urban health centers evaluating implementation of a primary care quality improvement strategy will likely not allow for generalizability to rural settings. We urge statistical reviewers to evaluate sampling procedures and watch for samples of convenience that may not be merited.

### Inclusion and Exclusion Criteria Appropriate for State of Knowledge

No matter the type or phase of study, the protocol should describe how eligibility of study participants is determined. The notion that therapies and diseases have differing underlying mechanisms of action or progression in different populations (e.g., children vs. adults, males vs. females) often leads to increased restrictions on inclusion and exclusion criteria. While sometimes justified scientifically as it allows for a precise estimate of effect within a specialized population, the tradeoff is less generalizability and feasibility to complete enrollment. On the other hand, sample selection or eligibility criteria may be expansive and purposefully inclusive to maximize generalizability. The tradeoff is often increased variability and potential heterogeneity of effect that within specific subgroups. Biostatistical reviewers should question eligibility decisions chosen purely for practical reasons and recognize the limits they place on a study’s generalizability, noting the potential future dilemma for managing patients who would not have completely satisfied a study’s inclusion and exclusion criteria.

### Screening and Enrollment Processes Minimize Bias and Do Not Restrict Diversity

It is imperative that clinical and translational research be designed for diversity, equity, and inclusion. Aside from specification of eligibility criteria, the specific way researchers plan to identify, recruit, screen, and ultimately enroll study participants may be prone to biases. For example, reading level and language of the informed consent document may impact accessibility. The timing and location of recruitment activities also restrict access both in person and by mail or electronic communication. Using email outreach or phone outreach to screen and identify patients will exclude those without easy access to technology or stable phone service. Some populations may prefer text messaging to phone calls; some may prefer messages from providers directly rather than participating study staff. Biostatistical reviewers should consider inclusive procedures and those appropriate for the target study population as they have potential to impact bias and variability within the sample. This can ameliorate or amplify both effect sizes and study generalizability.

## Measurements and Outcomes

### Choice of Measurements, Especially the Response Variable, Is Justified and Consistent with the Objectives

The statistical review should ensure that outcome measures are aligned with objectives and appropriately describe the response of the experiment at the unit of analysis of the study (e.g., participant, animal, cell). Outcomes should be clinically relevant, measured or scored on an appropriate scale, valid, objective, reliable, sensitive, specific, precise, and free from bias to the extent possible [13]. Statistically, the level of specification is important. As an example, risk of death can be assessed as the proportion of participants who die within a specified period of time (binary outcome), or as time to death (i.e., survival). These outcomes require different analytic approaches with consequences on statistical power and interpretation. Ideally, the outcome should provide the maximum possible statistical information. It is common for investigators to dichotomize continuous measurements (e.g., defining a treatment responder as a participant who achieves a certain change in the outcome rather than considering the continuous response of change from baseline). Information is lost when continuous and ordinal responses are replaced with binary or categorical outcomes, and this practice is generally discouraged [14–16]. If the biostatistical reviewer identifies such information loss, they should consider the resulting inefficiency (i.e., increased sample size required; loss of efficacy signal) in the context of the risks to human or animal subjects and the costs of the study.

Outcome deliberations become critical in the design of clinical trials because distinction between primary, secondary, and exploratory endpoints is important. Many considerations are the topic of a large body of literature [17–20]. The biostatistical reviewer should be aware that the Food and Drug Administration (FDA) differentiates among the decisions that are supported by primary, secondary, and exploratory endpoints, as described in the “Discussion of control of type I error (multiple comparisons) is present” section [18]. Results of primary and secondary endpoints must be reported in clinicaltrials.gov; results of exploratory endpoints do not require reporting. Regardless of the dictates of clinicaltrials.gov, limitations on the number of secondary endpoints are prudent, and all secondary endpoints should be explicitly detailed in the protocol [21]. It is important that the issue of multiplicity in endpoints is considered and clearly delineated in the sections on sample size and statistical analysis. Beyond multiplicity, what constitutes success of the trial needs to be clear. For example, if there are several co-primary endpoints, it should be noted whether success is achieved if any endpoint is positive, or only if all endpoints are met. It should also be clear whether secondary endpoints will be analyzed if the primary endpoint is not significant. When there are longitudinal measurements, the investigators should specify whether a single time point defines the primary endpoint or whether all time points are incorporated to define the trajectory of response as the primary endpoint. Although the description above focuses on clinical trials, all protocols should describe which outcome(s) is(are) the basis for sample size or integral to defining success of the study.

Composite outcomes deserve special attention in a biostatistical review of a protocol [22–24]. Composite outcomes combine several elements into a single variable. Examples include “days alive and out of hospital” or “death or recurrent myocardial infarction.” Investigators sometimes select composite outcomes because of a very low expected event rate on any one outcome. The biostatistical reviewer should carefully consider the information in the composite endpoint for appropriateness. An example of a composite outcome that would not be appropriate is death combined with lack of cognition (e.g., neurologically intact survival) when the causal pathway is divergent such that the treatment worsens mortality but improves cognitive outcome. Some patients will care about quality of life (i.e., improved cognition) over length of life, while others will not, which yields a situation where one cannot define a single utility function for the composite outcome.

### Timing of Assessments and Measurements Is Clear and Standardized (Study Schedule or Visit Matrix Should Be Present)

Regardless of whether a measurement is an outcome, predictor, or other measure, a biostatistical reviewer should evaluate each measurement in terms of who, what, when, where, how, and why, as shown in Table 2.

**Table 2.** Aspects of measurement that should be considered in protocol evaluation

• Who will be assessed?
• Who will make the assessments?
• Is the assessor blinded to the intervention arms?
• What is (are) the measurement variable(s)?
• What is the analysis metric (e.g., change from baseline, end of study value, time to event)?
• Where will the assessment take place (e.g., hospital, home, doctor’s office)?
• When will the assessments take place (specific time points)?
• How are the assessments summarized (e.g., mean, median, proportion)?
• How will the measurements be used in the analysis?
• Why are the assessments clinically relevant for addressing efficacy and safety outcomes?

This information tends to be scattered throughout the protocol: who will be assessed is described in the eligibility criteria; the main predictor may be defined in interventions; what and when are given in the outcomes section; who will take assessments, where and when are described in data collection methods; and how the assessments are summarized may be in the outcomes or the statistical methods section. This makes the evaluation of measurements challenging, but it is a vital component and should not be undervalued. A schedule of evaluations (Table 3), or visit matrix, can be a very valuable tool to summarize such information and help reviewers easily identify what measurements are being made by whom, how, and when.

**Table 3.** Schedule of evaluations

	Screening/baseline		Follow-up (FU) assessments		
Assessment procedure	Visit 1	Visit 2	FU 6	FU 12	FU 18
Participant consent	X				
HIPAA authorization form	X				
Personal information (demographics)	X				
Medical history		X			
Current medication use	X				
Primary outcome		X	X	X	X
Secondary outcomes		X	X		X
Expensive secondary outcome		X			X
Tertiary outcome		X	X	X	X
Blood collection		X	X		X
SF-36		X	X	X	X

### Objectively Measured and Standardized

To remove potential sources of bias, from a statistical perspective each assessment should be as objective as possible. Thus, the protocol should mention measures of standardization as appropriate (e.g., central reading of images, central laboratory processing), and measures to ensure fidelity and quality control. For example, if outcomes come from a structured interview or clinical rating, there should be discussion of measuring interrater agreement. If the agreement is lower than expected, training or re-training procedures should be described.

### If Based on Subjective or Patient Report, Use Validated Instruments as Appropriate

For patient-reported outcomes and questionnaires, the validity and reliability of the instrument is key. Validated instruments with good psychometric properties should be favored over unvalidated alternatives. The biostatistical reviewer should generally be cognizant of the repercussions of even small changes to the instrument, including reformatting or digitizing instruments.

### Measurements Are of Maximum Feasible Resolution with No Unnecessary Categorization in Data Collection

Reviewers should assess whether appropriate data collection methods are used to improve the quality of the outcomes. For example, outcomes sometimes require derivation or scoring, such as body mass index (BMI). The reliability of BMI is improved if height and weight are collected and BMI is calculated in analysis programs. This reduces errors in translating between feet and centimeters or incorrect calculations. For each key variable, it should be clear how the data will be generated and recorded; when a case report form is provided for review this can be remarkably helpful.

### Ranges of Outcomes, Distributional Properties, and Handling in Analyses Are Clear

The distributional properties of outcomes will allow a statistical reviewer to determine whether the analytic strategy is sound. If a laboratory value is known to be highly skewed or if the investigators use a count variable with several anticipated zero values, a standard t-test may not be appropriate, but rather nonparametric tests or strategies assuming a Poisson or zero-inflated Poisson distribution may be more appropriate. These will have implications on inferences and sample size calculations. Often, time-to-event variables are confused with binary outcome variables. Cumulative measures such as death by a certain time point (e.g., 12-month mortality rate) vs. time-to-death are two different outcomes that are incorrectly used interchangeably. The statistical reviewer should pay careful attention to situations where the outcome could feasibly be treated as binary (i.e., evaluated with relative risk, odds ratio, or risk difference) or as a time-to-event outcome (i.e., evaluated with hazard ratios).

### Algorithms Used to Derive Variables or Score Outcome Assessments Are Justified (e.g., Citations, Clinical Meaning)

Just as validated survey/questionnaires are preferred in study design, so are any algorithms that are being used to select participants, allocate interventions (or guide interventions), or derive outcomes. For example, there are several algorithms used to derive estimated glomerular filtration rate [25–27], a measure of kidney function, or percent predicted forced expiratory volume (FEV1), a measure of lung function [28, 29]. Some studies use predictive enrichment, using algorithms to select participants for inclusion. Although a statistical reviewer may not be in a position to argue the scientific context of the algorithm, they can ensure that the choice of algorithm or its derivation is discussed in the protocol. This should include whether investigators plan to use unvalidated algorithms or scores. If the algorithms are described according to the Transparent Reporting of multivariable prediction model for Individual Prognosis Or Diagnosis criteria [30], it provides the biostatistical reviewer the necessary information to either advocate for or against the algorithm as a component of the study.

### Measurement of Important/standard Explanatory Variables that Will Describe Sample or Address Confounding

In addition to outcomes, other measures such as predictors, confounders, effect modifiers, and other characteristics of the population (e.g., concomitant medications) should be listed. For example, if obesity is a confounder and will be included in analyses, the metric used to define obesity should be provided. These variables generally do not need as much detail as the outcome unless they are important in the analyses, or the lack of explanation may cause confusion.

## Treatment Assignment

### Minimization of Biases (e.g., Randomization and Blinding)

Any study that evaluates the effect of a treatment or intervention must consider how participants are allocated to receive the intervention or the comparator. Randomized assignments serve as the ideal study design for minimizing known and unknown differences between study groups and evaluating causality. With this approach, the only experimental condition that differs in comparing interventions is the intervention itself. A biostatistical reviewer should consider the fundamental components of the randomization process to ensure that threats to causal inference are not inadvertently introduced.

Units and methods of randomization will depend upon the goals and nature of the study. Units of randomization may be, for example, animals, patients, or clinics. There is a wide range of methods for controlling balance across study arms [31–33]. Simple randomization and block randomization are straightforward, but techniques such as stratification, minimization, or adaptive randomization may be more appropriate. The choice of randomization approach, including details of the randomization process, should be considered by the biostatistical reviewer in the context of the study design. The algorithm used to generate the allocation sequence should be explained (e.g., stratified blocks, minimization, simple randomization, response-adaptive, use of clusters). However, the reviewer should not ask for details that would defeat the purpose of concealment (e.g., size of blocks).

The integrity of a trial and its randomization process can easily be compromised if the allocation sequence is not concealed properly, and thus the biostatistical reviewer should look for a description of the concealment process, such as use of a central telephone system or centralized web-based system. Concealment is not the same as blinding (sometimes also referred to as masking); concealment of the randomization sequence is intended to prevent selection bias prior to enrollment whereas blinding is intended to prevent biases arising after enrollment. Therefore, open-label and non-blinded randomized studies should also conceal the allocation sequence. If a pre-generated sequence is not used, the biostatistical reviewer should consider how real-time randomization is deployed, as might be required in a response-adaptive design, and whether it is feasible.

Beyond considerations of generating and concealing the randomization algorithm, the biostatistical reviewer should look for biases arising from the randomization process. As an example, if there is extended time between randomization and intervention, there is a high likelihood that the participant’s baseline status has changed, and they may no longer be eligible for treatment with the intervention. This can result in an increased number of patients dropping out of the study or not receiving their assigned intervention. Under the intent-to-treat principle, this results in bias toward the null.

The use of blinding can strengthen the rigor of a study even if the participant’s treating physician cannot be blinded in the traditional sense. For example, a blinded assessor of the primary endpoint can be used. Sometimes blinding of the participant or physician is not possible, such as when intervention is a behavioral therapy in comparison to a medication. It may also be necessary to break the blind during the study in emergency cases or for study oversight by a Data and Safety Monitoring Board. The biostatistical reviewer should consider whether sufficient blinding is in place to minimize bias, and whether the process for maintaining and breaking the blind is sufficient to prevent accidentally revealing treatment allocation to those who may be positioned to introduce bias.

### Control Condition(s) Allow for Comparability or Minimization of Confounding

Randomization and blinding are used in prospective interventional trials to minimize bias and maximize the ability to conclude causation of the intervention. However, in some cases randomization may be unethical or infeasible and prospective observation is proposed to assess the treatment effect. In other cases, retrospective observational studies may be proposed. In such cases, there are several biases to which a biostatistical reviewer should be attuned. These include, but are not limited to, treatment selection bias, protopathic bias [34], confounding by severity, and confounding by indication. Approaches such as multiple regression methods or propensity score matching can be used to address measurable biases, and instrumental variable analysis can address unmeasured bias. The biostatistical reviewer should rightly negate the impact of an observational study of treatment effects when there is no attempt to mitigate the inherent biases, but should also support a protocol when appropriate methods are proposed.

## Data Integrity and Data Management

### Data Capture and Management Is Described

Data integrity is critical to all research. A biostatistical reviewer should be concerned with how the investigator plans to maintain the accuracy of data as they are generated, collected, and curated. Data collection and storage procedures should be sufficiently described to ascertain the integrity of the primary measurements and, if appropriate, to adjudicate compliance with regulatory and scientific oversight requirements. The amount of detail required is often proportional to the size and complexity of the research.

For larger and more complex studies, and clinical trials in particular, a standalone Data Management Plan (DMP) might be used to augment a research protocol [35, 36]. Whether the DMP is separate from the protocol, a biostatistical reviewer should consider details about who is responsible for creation and maintenance of the database; who will perform the data entry; and who, how, and when quality checks will be performed to ensure data integrity. This work is often supported by a data management platform, a custom system used to manage electronic data from entry to creation of an analytic dataset. The wrong tool can undermine data integrity, and a biostatistical reviewer should examine the data management pathway to ensure the final dataset is an accurate representation of the collected data. Investigators should describe their chosen data management platform and how it will support the workflow and satisfy security requirements. The choice of platform should be scaled to the study needs and explained in the study protocol; for supporting complex clinical trials spanning multiple countries, the technical requirements of the data management platform can become extensive.

Studies often utilize data captured from multiple platforms that must eventually be merged with the clinical data for analysis. Examples include data from wearable mobile health technology (smart phones and wearable devices such as accelerometers and step counters), real-time data streams from inpatient data monitors, electronic health record data, and data from laboratory or imaging cores. If the protocol calls for multiple modes of data collection, the protocol should acknowledge the need for merging data source and describe how data integrity will be assured during linkage [e.g., use of Globally Unique Identifiers to identify a single participant across multiple data sources, and reconciliation of such keys during the study).

### Security and Control of Access to Study Data Are Discussed

While protection of privacy and confidentiality is traditionally the purview of privacy boards or IRBs, a biostatistical reviewer should ensure the protocol describes data security measures. Expected measures will include procedures for ensuring appropriate authentication for use, storage of data on secure servers (as opposed to a local computer’s hard disk or unencrypted flash drive), and accessibility of the data or data management system. In a clinical trial, for example, the data management system might need to be available 24 h a day, 7 days a week with appropriate backup systems in place that would pick up the workflow in the event of a major failure. Conversely, a chart review study might be supported sufficiently using a simple data capture form deployed in Research Electronic Data Capture [37, 38]. Increasingly, data and applications are being maintained on cloud-based systems with high reliability; security requirements also apply to cloud-based data storage.

### Data Validation, Errors, Query Resolution Processes Are Included

Missing and erroneous data can have a significant impact on the analysis and results, so the biostatistical reviewer should evaluate plans to minimize missing data and to check for inaccuracies. Beyond traditional clinical trial monitoring for regulatory and protocol compliance, the protocol should consider how to prevent data values outside of allowable ranges and data inconsistencies. A query identification and resolution process that includes range and consistency checks is recommended. For more complex studies, the biostatistical reviewer should expect the investigator to describe plans for minimizing missing [39] or low-quality data during study implementation, such as routine data quality reporting with corrective action processes. Inherent to more complex research protocols are the practical challenges that result in protocol deviations, including dose modifications, study visits that occurred outside of the prescribed time window, and missed assessments during a study visit. The biostatistical reviewer may expect the DMP to describe the approach to documenting such events and how they are to be considered in subsequent analyses.

## Statistical Analysis Plan

Statistical analysis plans provide a reproducible roadmap of analysis that can be very valuable for all studies [40, 41]. There are many components that could be included and these will vary on the study type and design [42–44]. A true pilot or feasibility study may not require a statistical analysis plan with the detail that would typically be required for an RCT. The objectives of such studies are often not to answer a particular research question, but to determine feasibility of study conduct. Such studies should not involve the testing of hypotheses, but they often involve quantitative thresholds for enrollment to inform the larger studies. If the analysis plan calls for hypothesis testing, the biostatistical reviewer may rightfully reject the approach as inappropriate. For large epidemiological registries, formal hypotheses may not have been developed at the time of design. Unlike feasibility studies, however, the biostatistical reviewer should expect to see the research team’s general approach to developing and testing hypotheses or modeling the data. The following sections outline key components that should be considered by biostatistical reviewers. Most statistical inference, and the focus of this article, is frequentist. We note that many questions can be better answered using Bayesian inference. Almost all of the points of this article apply equally well to a Bayesian study.

### Statistical Approach Is Consistent with Hypothesis and Objectives

Statistical analyses allow for inferences from study data to address the study objectives. If the analyses are misaligned, the upstream research question that it may address, although potentially important, will not be that which the researcher had originally sought to explore. The biostatistical reviewer should ensure alignment between the proposed analyses and (1) the research hypotheses, and (2) the study outcomes. For example, continuous outcomes should not be analyzed using statistical methods designed for the analysis of dichotomous outcomes, such as chi-squared tests or logistic regression. A misaligned analysis plan may have implications not just for missing the study objectives but for sample size considerations. A study involving a continuous outcome but which employs analysis of a binary outcome is generally less efficient and will require more experimental units. A biostatistical reviewer should identify such inefficiencies, particularly in studies that involve extensive resources or that involve risk to human subjects.

For studies with multiple objectives, the biostatistical reviewer should expect a detailed analysis plan for each primary outcome. Secondary outcomes should be described but might be grouped together based on outcome type. Exploratory outcomes might be discussed with fewer details.

### A Plan for Describing the Dataset Is Given

Describing the study sample is the first step in any analysis, and it allows those evaluating results to determine generalizability. Thus, any analytic plan should call for a description of the study sample – e.g., baseline characteristics of patients, animals, cells – regardless of importance for the primary study goals. The description should include sampling, screening, and/or randomization methods, as in a Consolidated Standards of Reporting Trials (CONSORT) diagram [43] for clinical trials (see also Mathilde et al. [45] for other study types). When a study design involves repeated measurements on the same experimental unit (e.g., patient, animal, or cell), the biostatistical reviewer should consider each experimental unit’s contribution to the analysis at each time point.

### Unit of Analysis Is Clearly Described for Each Analysis

Experimental units may be clinical sites, communities, groups of participants, individual participants, cells, tissue samples, or muscle fibers. They may vary by research aim within a single study. A biostatistical reviewer’s evaluation of the analytical approach and sample size estimates depends on experimental unit. In cluster-randomized trials, for example, the analytic unit may be the cluster (e.g., clinic or site) or unit within a cluster (e.g., patients). Common mistakes in cluster-randomized trials, or studies involving analytic units that are inherently correlated with one another, involve failure to specify the units of analyses and failure to adequately account for the intraclass (or sometimes termed intracluster) correlation coefficient (ICC) in both sample size calculations and analyses [46, 47]. The biostatistical reviewer should consider whether the analytic strategy adequately accounts for potential correlation among experimental units in such studies.

### Analysis Populations Clearly Described (e.g., Intention-to-Treat Set, Per Protocol Set, Full Analysis Set)

Non-adherence or data anomalies are inevitable in clinical research, but decisions to exclude participants or data points from analyses present two problems: they result in a smaller overall analytic sample size, and they introduce a potential source of bias. Many large, clinical trials employ the intention-to-treat principle. Under this principle, once participants are randomized, they are always included in the analysis, and participants are analyzed as originally assigned regardless of adherence. Study protocols should mention any plans to analyze participants according to this principle and any modifications of this principle. As may occur with a safety analysis for an experimental drug or treatment, if an analysis assigns patients to treatment arms based on what actually happened (i.e., a *per protocol* or *as treated* dataset), the biostatistical reviewer should ensure this is pre-defined. That is, the criteria that make a participant “adherent” should be clear, including how adherence will be measured (e.g., pill counts, diaries). The biostatistical reviewer should also assess handling of non-adherent participants. The analysis plan should discuss these ideas and describe the analytic dataset with these questions in mind. These same concepts can be applied to observational studies to reduce bias and variability in analyses while keeping true to the study’s aims. Whether it be in the context of the study population in an RCT or in determining causality in an observational context, the biostatistical reviewer should also consider whether causal inference methodology is an appropriate approach to addressing research aims.

### Key Statistical Assumptions Are Addressed

Soundness of statistical analyses depends on several assumptions. It would be impractical to list all assumptions in a protocol, but the biostatistical reviewer should evaluate plans to check major assumptions, such as normality and independence, noting that sometimes specific assumptions may be relaxed depending upon study scenario. On the other hand, certain proposed methods may require clear articulation of “out-of-ordinary” or strong assumptions for them to be truly valid in a given context (e.g., the many assumptions that surround causal inference methods). An example of an appropriate way to acknowledge and plan for addressing model assumptions is a high-level statement: “We will assess the data for normality [with the appropriate methods stated here], transform as needed, and analyze using either Student’s t-test or the nonparametric equivalent as appropriate [again stating at least one specific method here].”

### Alternative Approaches in the Event of Violations of Assumptions Are Present

While impossible to foresee all possible violations of assumptions and thus plan for all possible alternative approaches that may be appropriate, the investigator should have a contingency plan if violations of assumptions are likely. A strong analysis plan will mention how it will be updated using appropriate version control to address each shift in approach; this documentation will allow for greatest transparency in any unexpected changes in analyses.

### Discussion of Control of Type I Error (Multiple Comparisons) Is Present

When an analysis plan calls for many statistical tests, the probability of making a false positive conclusion increases simply due to chance. The biostatistical reviewer should balance the possibility of such type I errors with the strength and context of inferences expected. For clinical trials designed to bring a drug to market, controlling type I error is extremely important and both the FDA and the European Medicines Agency have issued guidance on how to handle this [18]. For purely exploratory studies, controlling the type I error may be less important, but the possibility of false discovery should be acknowledged.

### Description of Preventing and Handling Missing Data Is Given

Missing data are often inevitable, especially in human studies. Poor handling of the missing data problem can introduce significant biases. The analysis plan should discuss anticipated missing data rates, unacceptable rates of missing data, and those that would merit exploration of in-depth sensitivity analyses.

If data are missing completely at random, analyses are generally unbiased, but this is very rarely the case. Under missing at random and missing not at random scenarios, imputation or advanced statistical methodology may be proposed, and the statistical reviewer should expect these to be clearly explained. If there are sensitivity analyses involving imputations, these also require explanation. While it is impractical to anticipate all possible missingness scenarios *a priori*, the biostatistical reviewer should at minimum determine (1) whether the protocol mentions anticipated missing data rate(s), (2) whether the anticipated rate(s) seem reasonable given the scenario and study population, (3) whether any missingness assumptions are merited, and (4) whether the analysis plans for imputation to explore multiple scenarios, allowing for a true sensitivity analysis.

### Interim Analyses and Statistical Stopping Guidelines Are Clear and Justified

The term interim analysis often signifies simple interim descriptive statistics to monitor accrual rates, process measures, and adverse events. A study protocol should pre-specify plans for interim data monitoring in this regard, but there are seldom statistical implications associated with these types of analyses. A biostatistical reviewer should pay more attention when the protocol calls for an interim analysis that involves hypothesis testing. This may occur in clinical trials or prospective studies that use interim data looks to make decisions about adapting study features (such as sample size) in some way, or to make decisions to stop a study for either futility or efficacy. If the study calls for stopping rules, the criteria should be pre-specified in the study protocol. These may be in the form of efficacy or safety boundaries, or futility thresholds [48–50]. The biostatistical reviewer should note that to control the type I error rate for stopping for benefit, an interim analysis for these purposes may necessitate a more conservative significance level upon final statistical analysis. The protocol should ideally state that no formal interim analyses will be conducted or explain the terms of such analyses to include the timing, the frequency or total number of interim “looks” planned, and approach to controlling type I and type II errors.

## Sample Size Justification

### Type I and II Error Rates Present for All Sample Size Calculations and Corresponding Statistical Tests

It is common for investigators to use conventional values of type I error rate (*α* = 0.05) and power (80%). However, there may be situations when more emphasis is put on controlling the type II error or the type I error. Phase II studies often aim to determine whether to proceed to a phase III confirmatory study rather than to determine whether a drug is efficacious. In this case, a significance level of 0.20 might be acceptable. For a large, confirmatory study, investigators focus on controlling the type I error, and it may be appropriate to set desired power to 90% or the significance level to 0.01. The biostatistical reviewer should evaluate the selected power and significance levels used to justify the sample size, including decisions to deviate from convention.

### Parameter Assumptions Are Clearly Stated and Justified (i.e., Based on Previous Research and Considers the Population Studied)

All power and sample size calculations require *a priori* assumptions and information. The more complicated the analyses, the more parameter assumptions that the investigators must suggest and justify. The statistical reviewer should be able to use the parameter assumptions provided in any proposed study to replicate the sample size and power calculations (at least to an approximate degree). The justification of the assumed parameter values (e.g., median time-to-death, variance estimates, correlation estimates, control proportions, etc.) should be supported by prior studies or literature.

Additionally, common issues in research such as attrition, loss to follow-up, and withdrawal from the study can greatly affect the final sample size. Sample size justifications usually account for these issues through inflating enrollment numbers beyond the sample size required to achieve the desired significance level and power. This will help ensure analytic sample size(s), after accounting for attrition and loss, resemble the required sample size as determined in the *a priori* calculations.

### Statistical Tests used in Sample Size Calculations Match Those Presented in Statistical Analysis Plan or Appropriately Justify Reasoning for Straying from It

It is critical that the statistical methods assumed for computing power or sample size match, as closely as possible, those that are proposed in the statistical analysis plan. If the primary statistical analysis methods are based on outcomes with continuous data, such as using a two-sample t-test, then the sample size justification should also assume the use of the two-sample t-test. If the primary statistical analysis methods are based on outcomes with categorical data, such as using the chi-squared test to compare proportions, then the sample size justification should also assume the use of the chi-squared test. A mismatch between the power and sample size calculations and the statistical approach essentially render the estimations uninformative. Even making assumptions about how the data are likely to be analyzed in practice, a biostatistical reviewer may barely be able to infer even gross accuracy of the estimates. In general, it may be acceptable to plan for a complicated analysis [e.g., an analysis of covariance, adjusting for baseline), but sample size considerations may be based on a simpler statistical method (e.g., a t-test). However, when there is sufficient information to replicate the data generation mechanism, simulation presents a straightforward solution to understanding the effect of design decisions on the sample size and is desirable when the inputs can be justified.

### Minimum Clinically Important Differences or Required Precision Described

Beyond the statistical approach, the factors that have the most influence on the sample size calculation are the minimally important difference between interventions (or change) and the variability of the primary outcome variable. When the minimally important difference is put in context of variability, the “effect size” can be estimated, and this drives the sample size justification. Investigators may propose the minimally important difference based upon clinically meaningful differences or based upon biologically useful differences. When a protocol proposes a minimally important difference arbitrarily or based on observations in preliminary studies, then the biostatistical reviewer should expect some justification that this quantity is biologically relevant and that it will help advance knowledge or learning in the specific research area.

As with the minimally important difference, the protocol should carefully justify the expected distribution of the primary outcome(s). Preliminary studies may provide estimates of the distribution, but preliminary studies often include only small samples in very controlled settings. These samples may not represent the heterogeneity of the population of interest. Estimates of effect sizes or variability from the published literature may also be suspect for multiple reasons, including the use of populations different to that of the present study and publication bias. When investigators rely on estimates from these studies, it may result in a plan for a smaller sample size than is actually required to find the minimally important difference.

The biostatistical reviewer should recognize that the nature of sample size calculations is inherently approximate, but expect the study investigators to be realistic in estimating the parameters used in the calculations. It is helpful when the investigator provides a table or figure displaying ranges for these parameters, the power, the significance level, and the sample size. This can be especially important for less common or novel study designs that may require additional parameter assumptions and consideration, such as the use of the ICC to inflate the sample size to account for clustering or site effects; special considerations for the selection of *a priori* estimates of standard deviations or proportions; clearly stated parameters for the margins of non-inferiority (for non-inferiority studies) or equivalence (for equivalence studies) [51]; and an accounting of potential interaction effects of interest between confounding variables.

#### Powering for subgroup analysis

Funders and regulatory agencies increasingly require investigators explore treatment effects within subgroups. It is impossible to power a study to detect the minimally important effect in every possible subgroup, but it might be reasonable to power the study to detect interactions between treatment effect and some subgrouping variables, as might be done for testing heterogeneity of treatment effects. More frequently, subgroup analyses may be considered exploratory. In this case, the sample size required to observe a minimally important difference within a subgroup is of less importance; however, one may expect some discussion of the magnitude of difference that might be observed within the subgroup. Biostatistical reviewers should expect to see additional considerations if subgroup analyses are planned.

## Reporting and Reproducibility

### Plans for Data Sharing and Archiving Are Present

Many United States Federal agencies, including the NIH, now require sharing of data on completion of the research. The protocol should describe the approach to sharing data publicly in accordance with governing rules. This process can be challenging as the de-identification of data is not trivial. Providing data in a manner that encourages secondary use requires attention to the processes for gaining access and for managing and supporting the requests. The biostatistical reviewer is well positioned to comment on the investigators plans for curating the final dataset for public use.

### Version Control or a Means of Ensuring Rigor, Transparency, Reproducibility in Any Processes Is Evident

Every change in research protocols, analysis plans, and datasets is an opportunity for error. Protocols that specify the process for version control and change management are generally more rigorous and reproducible than those that do not.

### Plan to Report Results According to Guidelines or Law

With the increased emphasis on transparency of research, there is a growing mandate to publicize clinical research in open databases, such as clinicaltrials.gov. While this is primarily a regulatory concern, a biostatistical reviewer should be cognizant of the effort required and timelines imposed for such reporting and expect this to be reflected in the protocol timeline and, if appropriate, budget.

### The Biostatistical Reviewer’s Additional Responsibility

Berger and Matthews stated that “Biostatistics is the discipline concerned with how we ought to make decisions when analyzing biomedical data. It is the evolving discipline concerned with formulating explicit rules to compensate both for the fallibility of human intuition in general and for biases in study design in particular” [52]. As such, the core of biostatistics is trying to uncover the truth. While some scientists are implicitly biased in believing the alternative hypothesis to be true, a biostatistician’s perspective is appropriately “equipoise.” For example, at the root of basic frequentist statistical hypothesis testing lies the assumption that the null hypothesis (which is often of least interest to investigators in a field) is true. This perspective may lead to viewing the biostatistician in a reviewer role as a skeptic, when in reality they are necessarily neutral. This makes the biostatistician’s perspective helpful and often imperative in protocol review. As an impartial reviewer and according to the foundations of a biostatistician’s education and training, it is therefore the biostatistician’s responsibility to (1) ensure sound study design and analyses, and to (2) be critical and look for flaws in study design that may result in invalid findings. Other content-specific reviewers may have a tendency toward overly enthusiastic review of a research study given the scientific significance of the proposed research or lack of viable treatment options for an understudied disease. The biostatistician reviewer thus often provides a viewpoint that is further removed and more impartial, with the responsibility to preserve scientific rigor and integrity for all study protocols, regardless of significance of the research. We note that a complete, impartial review may not always warrant the same level of feedback to investigators. For example, investigators submitting a grant for review will benefit from the direction of a thorough written critique with guidance. However, for an institution considering joining a multicenter protocol, the statistical review may simply be a go/no-go statement.

As biostatistical reviewers tend to possess both specialized quantitative training and collaborative experiences, exposing them to a broad range of research across multiple disciplines, we view the biostatistician reviewer as an essential voice in any protocol review process. Biostatisticians often engage collaboratively across multiple research domains throughout the study lifecycle, not just review. Given this breadth and depth of involvement, a biostatistician can contrast a proposed study with successful approaches encountered in other disciplines. The biostatistician thus inherits the responsibility to cross-fertilize important methodologies.

A biostatistical reviewer, with sound and constructive critique of study protocols prior to their implementation, has the potential to prevent issues such as poor-quality data abstraction from medical records, high rates of loss to follow-up, lack of separation between treatment groups, insufficient blinding, failure to cleanly capture primary endpoints, and overly optimistic accrual expectations, among other preventable issues. Protocol review offers a chance to predict many such failures, thereby preventing research waste and unnecessary risks.

In this article, we have discussed components of a study protocol that a biostatistical reviewer (and, indeed, all reviewers) should evaluate when assessing whether a proposed study will answer the scientific question at hand. We posit that the biostatistical reviewer, through their breadth of engagement across multiple disciplines and experience with a broad range of research designs, can and should contribute significantly beyond review of the statistical analysis plan and sample size justification. Through careful scientific review, including biostatistical review as we outline here, we hope to prevent excess resource expenditure and risk to humans and animals on poorly planned studies.
